# Gender Difference on the Effect of Omega-3 Polyunsaturated Fatty Acids on Acetaminophen-Induced Acute Liver Failure

**DOI:** 10.1155/2020/8096847

**Published:** 2020-08-27

**Authors:** Yunzhi Liu, Yu Chen, Xinghuan Xie, Aiping Yin, Yue Yin, Yan Liu, Lijun Dong, Zhengyumeng Zhu, Jia Zhou, Qingchun Zeng, Xiao Lu, Zhengliang Chen, Kun Wen, Daming Zuo

**Affiliations:** ^1^Institute of Molecular Immunology, School of Laboratory Medicine and Biotechnology, Southern Medical University, Guangzhou, Guangdong 510515, China; ^2^Guangdong Province Key Laboratory of Proteomics, Department of Immunology, School of Basic Medical Sciences, Southern Medical University, Guangzhou 510515, China; ^3^Key Laboratory for Organ Failure Research, Department of Cardiology, Nanfang Hospital, Southern Medical University, Guangzhou, Guangdong 510515, China; ^4^Division of Laboratory Medicine, Zhujiang Hospital, Southern Medical University, Guangzhou, Guangdong 510282, China; ^5^Microbiome Medicine Center, Zhujiang Hospital, Southern Medical University, Guangzhou, Guangdong 510282, China

## Abstract

Acetaminophen (APAP) toxicity is the leading cause of drug-induced liver failure, which is closely related to mitochondrial dysfunction and oxidative damage. Studies in clinical trials and in animal models have shown that omega-3 polyunsaturated fatty acids (n-3 PUFAs) affect the progression of various types of liver damage. Interestingly, the sex-dependent effect of n-3 PUFAs on human health has also been well documented. However, it is unknown whether supplementation of n-3 PUFAs modulates the pathogenesis of APAP-induced liver failure with sex-specificity. Our results showed that both endogenous and exogenous n-3 PUFAs significantly aggravated the APAP-induced liver injury in male mice, whereas the opposite effects were observed in females. In vivo and in vitro studies demonstrated that estrogen contributes to the gender difference in the regulation of n-3 PUFAs on APAP overdose. We found that n-3 PUFA-mediated regulation of hepatic oxidative stress response and autophagy upon APAP challenge is distinct between male and female mice. Moreover, we provided evidence that *β*-catenin signaling activation is responsible for the sex-dependent regulation of APAP hepatotoxicity by n-3 PUFAs. Together, these findings indicated that supplementation with n-3 PUFAs displays sex-differential effect on APAP hepatotoxicity and could have profound significance in the clinical management for drug-induced liver injury.

## 1. Introduction

Acetaminophen (APAP) is currently one of the most widely used antipyretic and analgesic drugs. Although this drug has been considered safe for decades, an overdose can cause severe liver damage, which ultimately may cause acute liver failure [1]. APAP hepatotoxicity involves its conversion to N-acetyl-p-benzoquinone imine (NAPQI), which consumes glutathione (GSH) and leads to the generation of reactive oxygen species (ROS). The accumulation of ROS contributes to sustained c-Jun N-terminal kinase (JNK) activation, which acts as determined signaling related to hepatocyte necrosis and apoptosis [2, 3]. It was reported that both inhibitors of ROS or JNK protected mice from APAP-induced liver injury [4, 5]. Autophagy is a cellular process that can degrade impaired mitochondria, thereby removing accumulated ROS [6]. It has been confirmed that increased autophagy protects mice against APAP-induced liver failure [7, 8]. During autophagy, cytosolic microtubule-associated protein 1 light chain 3 (LC3-I) is conjugated with phosphatidylethanolamine (PE). The PE-conjugated form of LC3 (LC3-II) is then recruited to the autophagosomal membrane, leading to the formation of autophagosome [9]. p62 (SQSTM1) is one of the selective autophagy receptors, which directly binds to LC3, transporting ubiquitination-related protein accumulates to the autophagosome [10].

n-3 polyunsaturated fatty acids (n-3 PUFAs), mainly including eicosapentaenoic acid (EPA) and docosahexaenoic acid (DHA), are essential to human health [11]. In particular, studies have documented that n-3 PUFAs affect the progression of various liver diseases [12–16]. Mechanically, several studies have reported that n-3 PUFAs modulated the hepatic oxidative stress response and autophagy activation during liver injury [14, 16]. Interestingly, the sex differences in the effects of n-3 PUFAs on health and disease have been widely studied [17–20]. Previous researches have demonstrated the sex-dependent differences in the metabolism of n-3 PUFAs in both humans and animals [21, 22]. Phang et al. reported that the differential regulation of hemostasis by n-3 PUFA supplementation in men and women is significantly associated with the levels of sex hormones [20]. It is of note to mention that sex hormones altered the synthesis of n-3 PUFAs in human primary hepatocytes [23], and the effect of n-3 PUFAs on nonalcohol fatty liver disease- (NAFLD-) related liver injury also exhibited gender difference [19].

The *fat-1* mice, which express the *Caenorhabditis elegans fat-1* gene, are capable of endogenous conversion of n-6 PUFAs to n-3 PUFAs, resulting in elevated amounts of n-3 PUFAs in their organs and tissues compared with the wild-type (WT) littermates [24]. In this study, we aimed to investigate the effect of n-3 PUFAs on APAP-induced liver damage in male and female *fat-1* mice. The result showed that male *fat-1* mice were susceptible to APAP-induced liver injury compared to WT mice, while the female *fat-1* mice were resistant to the APAP toxicity compared to the WT counterparts. Male *fat-1* mice had a lower activation of autophagy and higher generation of ROS than WT mice upon the APAP challenge. In female mice, the *fat-1* mice displayed promoted autophagy activation and limited ROS production compared to WT controls in response to APAP overdose. We have demonstrated that *β*-catenin signaling activation is involved in the differential regulation of APAP hepatotoxicity by n-3 PUFAs in male and female mice. Furthermore, we also provided evidence that exogenous n-3 PUFAs modulated the APAP hepatotoxicity in a sex-dependent manner.

## 2. Materials and Methods

### 2.1. Animals

WT C57BL/6 mice were obtained from the Laboratory Animal Center of Southern Medical University (Guangzhou, China). *Fat-1* mice were backcrossed with C57BL/6 mice, and the *fat-1* genotype of each mouse was identified by PCR assay using isolated genomic DNA from mouse tails as previously described [25]. All animal experiments were approved by the Welfare and Ethical Committee for Experimental Animal Care of Southern Medical University.

### 2.2. Reagents

DHA (D2534) and *β*-estradiol (E8875) were purchased from Sigma-Aldrich (St. Louis, MO, USA). The primary antibodies of *β*-catenin (66379-1-Ig), GSK3*β* (22104-1-AP), and GAPDH (10494-1-AP) were obtained from Proteintech (Chicago, IL, USA). Antibodies against phospho-*β*-catenin (DF2989) and phospho-JNK (AF3318) were from Affinity (Ancaster, ON, Canada). Antibodies for JNK (9252) and phospho-GSK3*β* (D3A4) were purchased from Cell Signaling Technology (Danvers, MA, USA).

### 2.3. Induction of Liver Injury

APAP (Santa Cruz Biotechnology, Santa Cruz, CA, USA) was dissolved in phosphate-buffered saline. Mice (8 weeks old) were fasted overnight and injected with APAP intraperitoneally at the dose of 400 mg/kg to induce hepatotoxicity and at the dose of 600 mg/kg to monitor survival rate. The serum was collected for alanine aminotransferase (ALT) and lactate dehydrogenase (LDH) assay. The tissue homogenates from the mouse liver were used to evaluate the levels of GSH with a commercial kit (Jiancheng Biotech, Nanjing, China). Liver damage was detected by hematoxylin and eosin (H&E) staining. Cell death was evaluated by TUNEL staining (Beyotime Biotechnology, Shanghai, China) according to the manufacturer's instruction. In some cases, male WT and *fat-1* mice were injected with 100 mg/kg *β*-estradiol (E2) intraperitoneally 1 week before APAP administration. To test the effect of exogenous n-3 PUFAs on APAP toxicity, male and female WT mice were fed with an n-3 PUFA-enriched diet 3 weeks before APAP injection.

### 2.4. Cell Culture and Treatment

HepaRG cells were obtained from Biopredic International (Rennes, France). The cells were cultured in William's E medium supplemented with 10% fetal bovine serum (FBS), 5 *μ*g/mL insulin, and hydrocortisone. The cells were stimulated with 20 mM APAP. In some cases, cells were pretreated with 50 *μ*M DHA, 2 *μ*M XAV939, or 100 nM E2.

### 2.5. Isolation of Hepatocytes

Hepatocytes were isolated as previously described [26]. Briefly, livers from APAP-treated WT or *fat-1* mice were perfused with the calcium-free salt solution through the portal vein, followed by perfusion with type IV collagenase *in situ*. The livers were then filtered with polyamide mesh. After centrifugation at 50 × g for 3 minutes, the supernatants were removed. The cells were used for flow cytometric assay or immunoblotting assay. For the culture of primary hepatocytes, cells were plated in 6-well plates coated with mouse tail collagen in William's E medium containing 10% FBS. After 4 hours of incubation, the culture was washed with phosphate-buffered saline and replaced with serum-free RPMI 1640 medium. Cells were incubated overnight before further treatment.

### 2.6. Flow Cytometric Assay

Annexin V/PI apoptosis kit was obtained from MultiSciences (Hangzhou, China). To measure the intracellular level of ROS, hepatocytes from APAP-treated WT and *fat-1* mice or APAP-stimulated HepaRG cells were incubated with 10 *μ*M DCFH-DA in the dark for 30 minutes at 37°C. The lipophilic cationic fluorescent dye JC-1 (KeyGEN, Nanjing, China) was used to detect changes in the mitochondrial membrane potential. The cells were acquired and analyzed using the BD FACSDiva program in the flow cytometry FACS LSRFortessa (BD Biosciences, San Jose, CA, USA).

### 2.7. Immunoprecipitation and Immunoblotting

Protein samples extracted from mouse liver or HepaRG cells were separated by SDS-PAGE and then transferred onto polyvinylidene fluoride (PVDF) membranes (Millipore, Billerica, MA, USA). The membranes were blocked by bovine serum albumin (BSA, 5%) for 1 hour at room temperature and incubated with indicated primary antibodies at 4°C overnight. Subsequently, the membranes were stained with horseradish peroxidase- (HRP-) conjugated corresponding secondary antibody. For immunoprecipitation, whole-cell lysates (WCLs) were incubated with antibodies (1 *μ*g) and protein A/G agarose (Santa Cruz Biotechnology) at 4°C overnight. Eluted immunoprecipitates were resolved on SDS-PAGE and examined for an association of proteins of interest using specific antibodies. Finally, measurement of the target protein was conducted with enhanced chemiluminescence (Thermo Fisher, Carlsbad, CA, USA) according to the manufacturer's protocol.

### 2.8. Immunohistochemistry and Immunofluorescence

After hydration, antigen retrieval was performed in citrate buffer (pH 6.0) at 100°C for 10 minutes, and endogenous peroxidase activity was blocked by incubation with 3% H_2_O_2_ for 15 minutes. After blocking with normal goat serum at 37°C for 1 hour, the sections were stained with the anti-*β*-catenin antibody at 4°C overnight followed by incubation with HRP-conjugated secondary antibody at 37°C for 1 hour. Visualization of the immunoreactivity was performed by enhanced diaminobenzidine kit (TransGen Biotech) and nuclear staining with hematoxylin.

For analysis of the colocalization of *β*-catenin with GSK3*β*, the HepaRG cells were fixed with 2% paraformaldehyde for 20 minutes and permeabilized with 0.1% Triton X-100 for another 5 minutes at room temperature. After blocking with 10% goat serum for 1 hour, cells were stained with primary antibodies overnight at 4°C followed by incubation with the corresponding fluorescent-labeled secondary antibodies for 1 hour at room temperature. The images were acquired with a ×63 oil immersion objective on an Olympus FV1000 confocal microscope (Shinjuku, Tokyo, Japan).

### 2.9. Statistical Analysis

The experimental data were expressed as mean ± SEM. One-way ANOVA was used to analyze the significant differences among multiple groups. Differences between two groups in the experiments were evaluated by Student's *t*-test. Comparison of the survival curves was determined using the log-rank test. Differences were considered statistically significant at *p* < 0.05.

## 3. Results

### 3.1. Endogenous n-3 PUFAs Exhibit the Sex-Dependent Manner in the Regulation of APAP-Induced Liver Injury

**Figure 1:**
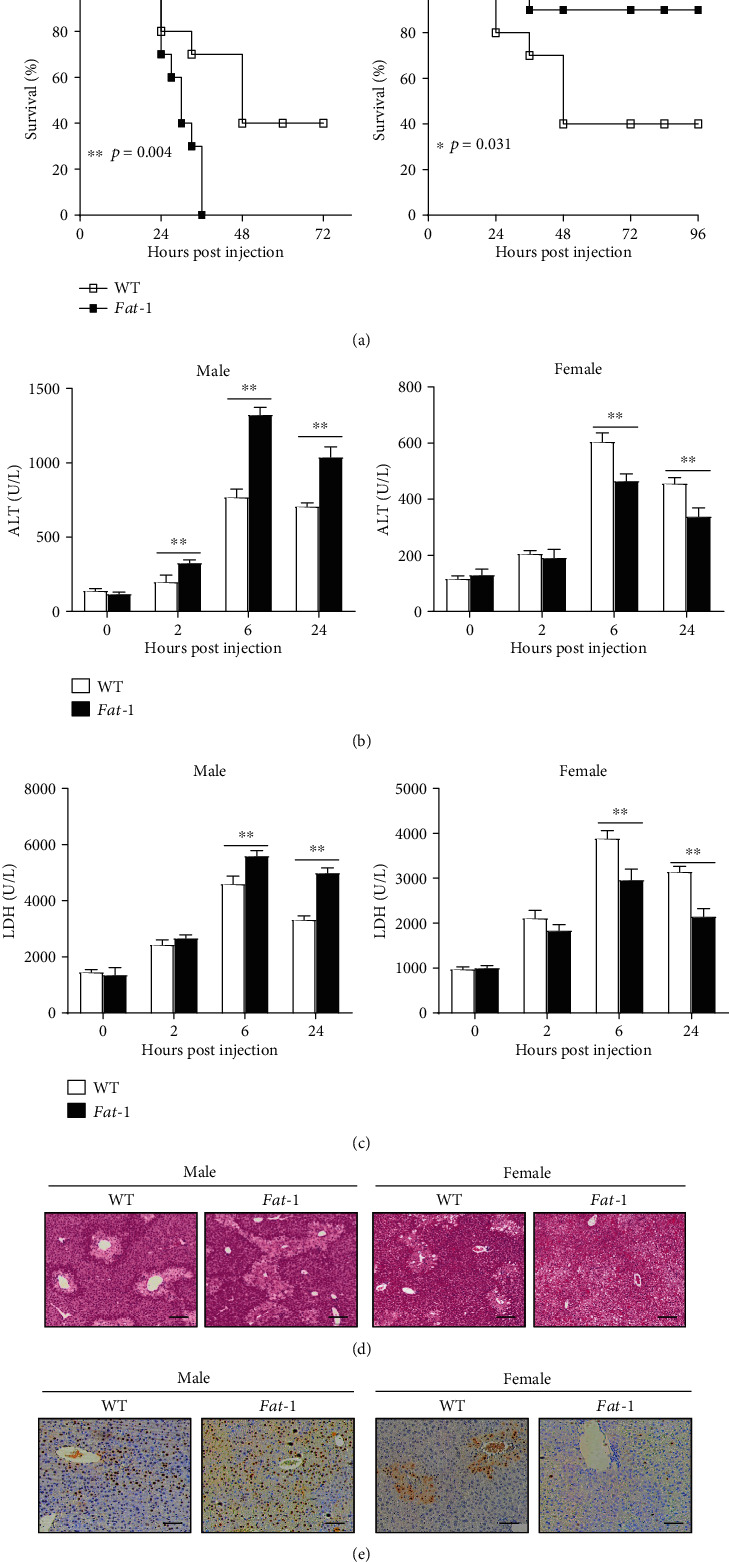
Endogenous n-3 PUFAs exhibit sex-differential effects on APAP-induced liver damage. (a) Male or female WT and *fat-1* mice (*n* = 10) were challenged with APAP at the dose of 600 mg/kg, and the survival of mice was monitored. (b–e) APAP (400 mg/kg) was intraperitoneally injected into male or female WT and *fat-1* transgenic mice (*n* = 5). (b, c) Serum ALT and LDH levels at different time points after APAP injection were measured. (d) Histological analysis of mouse livers was performed at 24 hours post-APAP injection by H&E staining. Scale bars = 100 *μ*m. (e) TUNEL staining was used to evaluate cell apoptosis in mouse livers. Scale bars = 50 *μ*m. ^∗^*p* < 0.05, ^∗∗^*p* < 0.01. One of the three independent experiments is shown.

To determine the effect of n-3 PUFAs on APAP-induced liver injury, sex- and age-matched WT or *fat-1* mice were challenged with a lethal dose of APAP. Surprisingly, the male *fat-1* mice exhibited higher mortality than male WT mice in response to APAP administration. However, the endogenous n-3 PUFAs significantly enhanced the survival rate in APAP-treated female mice (Figure 1(a)). To further evaluate the effect of n-3 PUFAs on APAP-induced liver injury, mice received a low dose of APAP, followed by biochemical and histological analyses. Consistently, elevated sera activities of ALT and LDH were observed in APAP-treated male *fat-1* mice compared to male WT controls, while sera from APAP-treated female *fat-1* mice displayed significantly decreased activities of ALT and LDH compared with those from female WT control mice (Figures 1(b) and 1(c)). Histological analysis showed that liver tissues from male *fat-1* mice exhibited much more centrilobular hepatic necrosis than those from male WT mice after APAP challenge (Figure 1(d)). By contrast, the histological changes were significantly reduced in APAP-treated female *fat-1* mice compared to the female WT counterparts (Figure 1(d)). Additionally, the TUNEL-positive cells were significantly higher in the livers of male *fat-1* mice than in those of male WT mice upon APAP challenge, while the number of TUNEL-positive hepatocytes in APAP-treated *fat-1* females was less than that in female counterparts (Figure 1(e)). Together, these data implicate that endogenous n-3 PUFAs aggravate APAP-induced liver injury in male mice but ameliorate APAP-induced liver injury in female mice.

### 3.2. Estrogen Contributes to the Gender Difference in the Effect of n-3 PUFAs on APAP-Induced Hepatotoxicity

**Figure 2:**
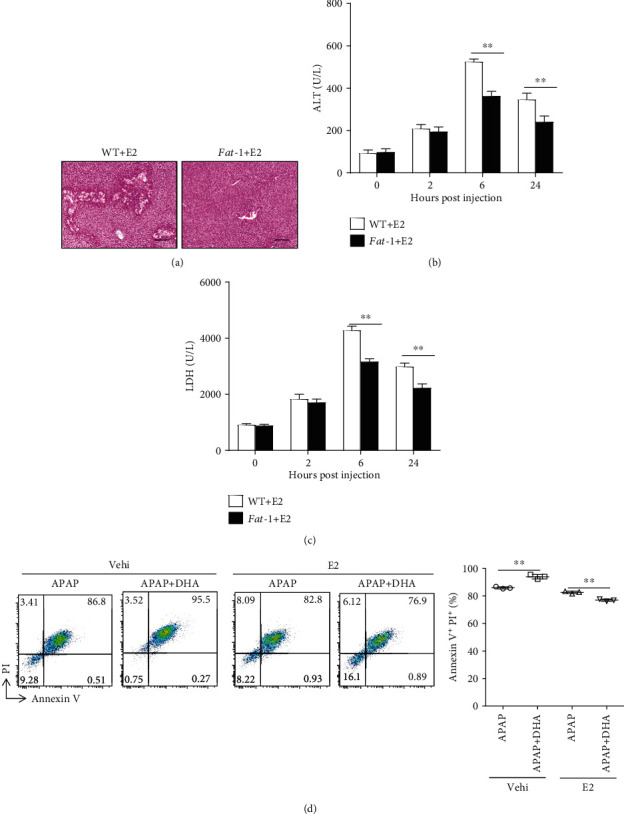
Estrogen is responsible for the sex-related function of n-3 PUFAs on APAP hepatotoxicity. (a–c) 100 mg/kg E2 was intraperitoneally injected into male WT or *fat-1* mice 7 days before 400 mg/kg APAP administration (*n* = 5). (a) 24 hours after APAP injection, histological analysis of livers was performed by H&E staining. Scale bars = 100 *μ*m. (b, c) Serum ALT and LDH levels in various time points after APAP administration was evaluated. (d) HepaRG cells were pretreated with 50 *μ*M DHA with or without 100 nM E2, followed by stimulation with 20 mM APAP for 24 hours. The cells were harvested and stained with Annexin V-FITC and PI for FACS analysis. ^∗∗^*p* < 0.01. The data represent three independent experiments with similar results.

We hypothesized that estrogen might play a critical role in determining the regulatory function of n-3 PUFAs on APAP-induced hepatotoxicity. Male WT and *fat-1* mice were injected with E2 one week prior to APAP administration. Interestingly, the hormone-treated *fat-1* mice exhibited milder liver damage than WT controls in response to APAP injection, as indicated by H&E staining (Figure 2(a)). Also, attenuated activities of ALT and LDH were found in sera obtained from E2-injected *fat-1* mice compared to those from WT counterparts after the APAP challenge (Figures 2(b) and 2(c)). Moreover, we evaluated the effect of estrogen on n-3 PUFA-mediated regulation of APAP toxicity in human HepaRG cells, a reliable model to study mechanisms of APAP hepatotoxicity in humans [27]. The result showed that DHA alone accelerated APAP-induced cell death, but DHA plus estrogen treatment strongly attenuated the APAP hepatotoxicity (Figure 2(d)). These results indicate that the hormonal factor is responsible for the sex difference in n-3 PUFA-modulated APAP hepatotoxicity.

### 3.3. n-3 PUFA-Mediated Regulation of Hepatic Oxidative Stress Response and Autophagy Is Distinct between Male and Female Mice

**Figure 3:**
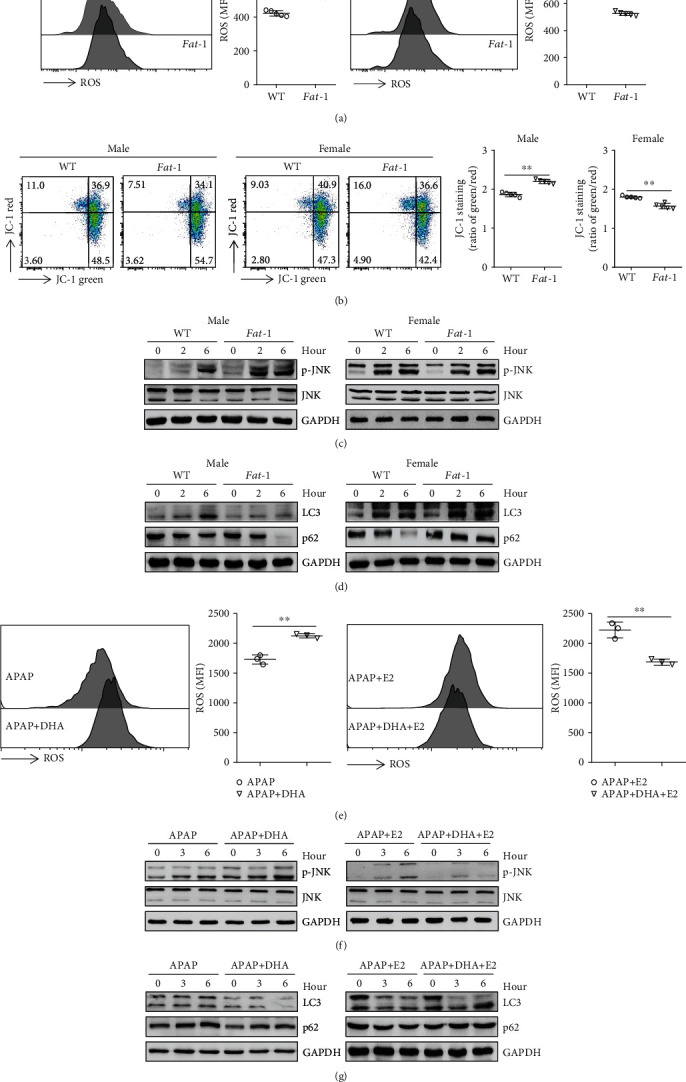
n-3 PUFA-modulated hepatic oxidative response and autophagy activation against APAP toxicity is distinct between male and female mice. (a–d) Male or female WT and *fat-1* mice were injected with 400 mg/kg APAP, and livers were collected at 6 hours post-APAP injection (*n* = 5). (a) Flow cytometry analysis of the intercellular ROS level by the fluorescent probe DCFH-DA in hepatocytes was carried out. (b) The mitochondrial membrane potential in hepatocytes was measured by the JC-1 dye staining for flow cytometry analysis. (c) Phosphorylation of JNK was evaluated by immunoblotting analysis at the indicated time after APAP challenge. (d) The protein levels of LC3 and p62 were examined by immunoblotting analysis. (e–g) HepaRG cells were pretreated with 50 *μ*M DHA with or without100 nM E2 prior to stimulation with 20 mM APAP. (e) The ROS level in the cells was analyzed by flow cytometry labeling with fluorescent probe DCFH-DA at 6 hours following APAP administration. (f) Phosphorylation of JNK expression in the APAP-treated cells was evaluated by immunoblotting analysis. (g) The hepatic levels of LC3 and p62 were determined by immunoblotting analysis. ^∗^*p* < 0.05, ^∗∗^*p* < 0.01. The data represent three independent experiments with similar results.

The APAP hepatoxicity is mainly due to the formation of NAPQI, which is generally detoxified by GSH [28]. Surprisingly, we found comparable GSH levels in the liver tissue homogenate from both APAP-treated WT and *fat-1* mice without sex difference (Supplemental Fig. 1A). Also, the hepatic GSH levels in APAP-injected WT and *fat-1* mice were similar after estrogen treatment (Supplemental Fig. 1B). ROS are critical mediators of APAP-induced cell death [29, 30]. Our result showed that hepatocytes from male *fat-1* mice contained much more intracellular ROS levels than those from WT males upon APAP challenge, which indicated that n-3 PUFAs might promote the oxidative stress during APAP treatment in male mice (Figure 3(a)). In contrast, a reduced ROS level was observed in the hepatocytes in the APAP-injected female *fat-1* mice compared to the female WT counterparts (Figure 3(a)). Given that mitochondria are a primary source of ROS in animal cells, we used JC-1 dye to evaluate the mitochondrial membrane potential by flow cytometry. The results indicated more damaged mitochondria in the hepatocytes from male *fat-1* mice than male WT counterparts, while there were less injured mitochondria in the liver cells from female *fat-1* mice compared with female WT controls, after APAP challenge (Figure 3(b)).

In hepatocytes, JNK plays an essential role in stress response and is activated by a diverse array of stresses, including oxidative stress [30]. We, therefore, investigated activation of the JNK pathway during APAP-induced liver injury. The results showed that APAP induced a strongly higher level of JNK phosphorylation in livers from male *fat-1* mice than those from male WT counterparts (Figure 3(c)). On the contrary, decreased JNK phosphorylation was observed in female *fat-1* mice compared to WT mice after the APAP challenge (Figure 3(c)).

Autophagy is involved in the clearance of damaged mitochondria, thereby ameliorating intracellular oxidative stress response during APAP treatment [7]. Upon APAP challenge, reduced LC3 and p62 expressions were observed in the liver obtained from male *fat-1* mice compared to that from male WT mice (Figure 3(d)). By contrast, elevated LC3 and p62 levels were found in the liver tissues from female *fat-1* mice compared with those from female WT controls after APAP injection (Figure 3(d)).

Next, we questioned whether estrogen modulates the n-3 PUFA-mediated regulation of APAP-induced oxidative stress and autophagy. DHA significantly enhanced the ROS production and JNK phosphorylation initiated by APAP stimulation in human hepatocytes. However, DHA downregulated APAP-triggered ROS production and JNK pathway activation in the presence of estrogen (Figures 3(e) and 3(f)). Consistently, DHA treatment reduced LC3 and p62 expressions in HepaRG cells upon APAP challenge, while increased LC3 and p62 levels were found in DHA-treated cells in combination with estrogen stimulation compared to the cells treated with estrogen alone (Figure 3(g)). Collectively, these data indicate that n-3 PUFA-modulated hepatic oxidative response and autophagy activation against APAP toxicity are sex-dependent.

### 3.4. Estrogen Mediates the Sex-Dependent Effect of n-3 PUFAs on APAP-Induced Hepatotoxicity via Regulation of *β*-Catenin Signaling

**Figure 4:**
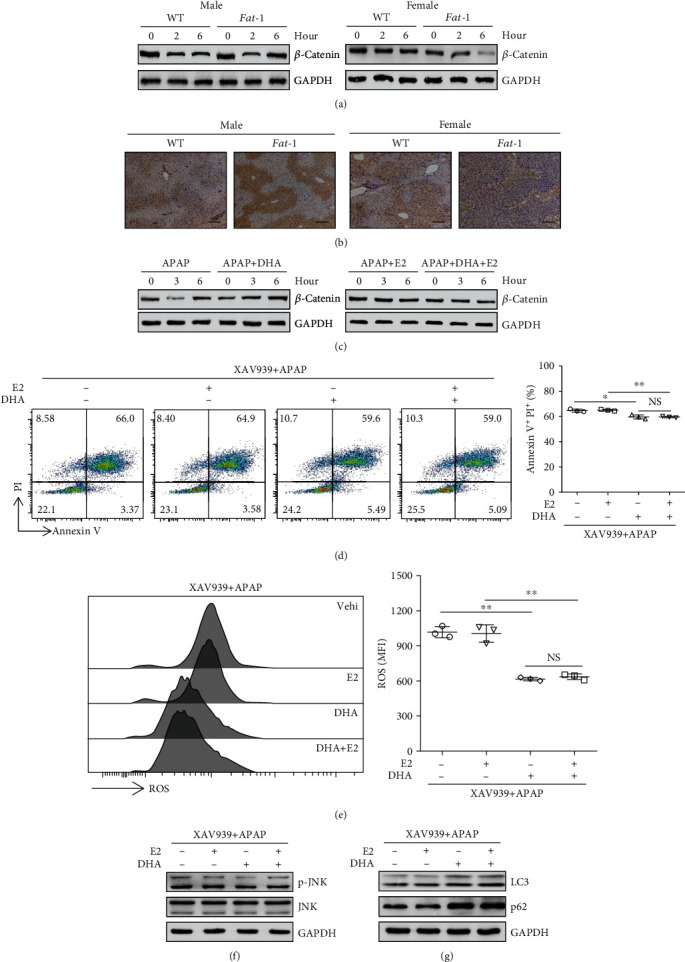
*β*-Catenin signaling is involved in the differential effect of n-3 PUFAs on APAP hepatotoxicity between male and female mice. (a, b) APAP (400 mg/kg) was intraperitoneally injected to male or female WT and *fat-1* mice (*n* = 5). Subsequently, the liver tissues were collected at 6 hours post-APAP injection. (a) The protein level of *β*-catenin in the liver tissues was evaluated by western blotting. (b) Immunohistochemical staining for *β*-catenin was determined in the liver tissue. Scale bars = 100 *μ*m. (c) HepaRG cells were pretreated with 50 *μ*M DHA without 100 nM E2 before stimulation with 20 mM APAP. Western blot assay of *β*-catenin was performed at the indicated time point after APAP stimulation. (d–g) HepaRG cells were pretreated with 2 *μ*M XAV939 for 2 hours combined with 100 nM E2 in the presence or absence of 50 *μ*M DHA. Subsequently, the cells were stimulated with APAP for another 24 hours. (d) Apoptosis was measured by Annexin V-PI staining, followed by FACS analysis. (e) The ROS level in the cells was analyzed by flow cytometry labeling with fluorescent probe DCFH-DA at 6 hours following APAP administration. (f) Phosphorylation of JNK expression in the APAP-treated cells was evaluated at 6 hours following APAP administration by immunoblotting analysis. (g) The expression of LC3 and p62 was determined by immunoblotting analysis. ^∗^*p* < 0.05, ^∗∗^*p* < 0.01. NS: not significant. The data represent three independent experiments with similar results.

Given that the *β*-catenin pathway is a negative modulator of autophagy and repressor of both LC3 and p62 expressions [31], we next investigated whether *β*-catenin signaling is involved in the differential effect of n-3 PUFAs on APAP hepatotoxicity between male and female mice. As determined by immunoblotting analysis and immunohistochemical staining, an elevated level of *β*-catenin was found in liver tissues from male *fat-1* mice compared with those from WT controls while livers from female *fat-1* mice exhibited decreased *β*-catenin expression compared to female WT mice after APAP injection (Figures 4(a) and 4(b)). Consistently, DHA significantly boosted *β*-catenin expression in hepatocytes during APAP exposure, whereas DHA plus estrogen treatment suppressed the expression level of *β*-catenin in cells compared to the cells treated with estrogen alone in response to APAP stimulation (Figure 4(c)).

To further confirm the role of *β*-catenin in n-3 PUFA-mediated regulation of APAP hepatotoxicity, HepaRG cells were pretreated with XAV939, a *β*-catenin inhibitor [32], prior to APAP stimulation. Surprisingly, APAP-induced cell death was similar in DHA only or DHA plus estrogen-treated cells with XVA939 pretreatment (Figure 4(d)). Similarly, XVA939 pretreatment abrogated the estrogen-mediated downregulation of ROS production and JNK phosphorylation in hepatocytes against APAP stimulation (Figures 4(e) and 4(f)). Moreover, the levels of LC3 and p62 were comparable in cells treated with DHA alone or with DHA combined with estrogen in the presence of XVA939 (Figure 4(g)). Furthermore, the primary hepatocytes from WT and *fat-1* mice were isolated and stimulated with APAP. In the presence of the *β*-catenin inhibitor, APAP-triggered ROS production was similar in the hepatocytes treated with or without estrogen (Supplemental Fig. 2A). Besides, the activation of the JNK pathway and the expressions of LC3 and p62 were comparable between the APAP-treated hepatocytes stimulated with or without estrogen, in the presence of XVA939 (Supplemental Fig. 2B and C). These data suggest that *β*-catenin signaling is responsible for the differential regulation of APAP hepatotoxicity by n-3 PUFAs in male and female mice.

### 3.5. GSK3*β* Is Involved in the Regulation of *β*-Catenin Signaling in Response to n-3 PUFAs

**Figure 5:**
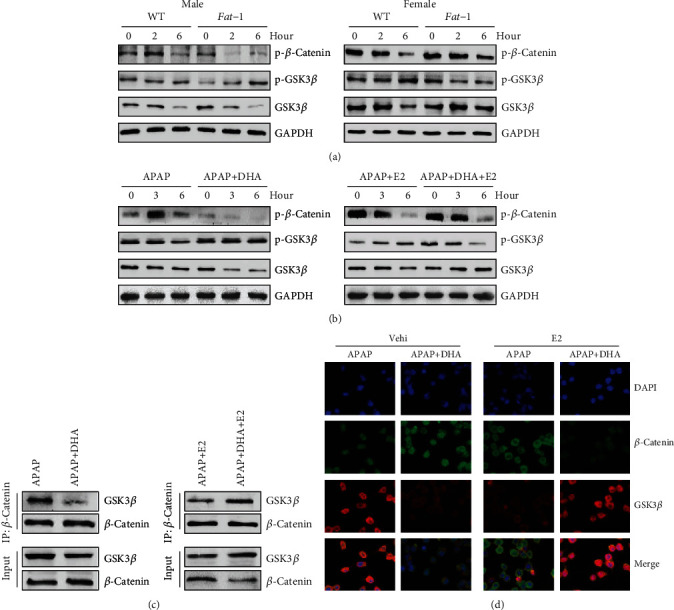
GSK3*β* contributes to the *β*-catenin signaling activation in response to n-3 PUFAs. (a) APAP (400 mg/kg) was intraperitoneally injected to male or female WT and *fat-1* transgenic mice (*n* = 5). The phosphorylated levels of *β*-catenin and GSK3*β* were determined by immunoblotting assay. (b–d) HepaRG cells were pretreated with 50 *μ*M DHA for 2 hours or 100 nM E2 for overnight before stimulated with 20 mM APAP. (b) The phosphorylation of *β*-catenin and GSK3*β* was determined by immunoblotting analysis at the indicated time point after APAP stimulation. (c, d) After being stimulated with APAP for 24 hours, the association of *β*-catenin with GSK3*β* in the cells was evaluated by immunoprecipitation (c) and immunofluorescence staining (d). The data represent three independent experiments with similar results.

Suppression of GSK3-mediated *β*-catenin phosphorylation is considered to be a critical event in Wnt/*β*-catenin signaling [33, 34]. Herein, increased phosphorylation of GSK3*β* and decreased phosphorylation of *β*-catenin were displayed in the livers from APAP-treated male *fat-1* mice compared with those from male WT controls (Figure 5(a)). On the contrary, the liver tissues from female *fat-1* mice exhibited much more reduced GSK3*β* phosphorylation and elevated *β*-catenin phosphorylation than those from female WT mice after APAP challenge (Figure 5(a)). An *in vitro* study demonstrated that DHA boosted GSK3*β* phosphorylation, thereby reducing the level of GSK3*β* and phosphorylated *β*-catenin in hepatocytes against APAP exposure (Figure 5(b)). However, DHA showed a reverse effect on the GSK3*β* phosphorylation and *β*-catenin signaling activation in APAP-stimulated cells in the presence of estrogen (Figure 5(b)). It has been reported that *β*-catenin is phosphorylated by GSK3*β* in a complex that consists of Axin, GSK3*β*, and *β*-catenin [34]. Immunoprecipitation assay showed that DHA treatment suppressed the interaction between *β*-catenin and GSK3*β* upon APAP challenge, while DHA promoted the binding of *β*-catenin with GSK3*β* in APAP-stimulated cells in the presence of estrogen (Figure 5(c)). Furthermore, immunofluorescence staining validated that DHA alone limited the interaction between *β*-catenin and GSK3*β*, while DHA plus estrogen enhanced the binding of *β*-catenin with GSK3*β* compared to estrogen alone in the APAP-stimulated cells (Figure 5(d)). These results suggest that GSK3*β* contributes to the sex hormone-dependent effect of DHA on *β*-catenin signaling activation.

### 3.6. Exogenous n-3 PUFAs Exhibit Similar Effect on APAP-Induced Liver Injury with Endogenous n-3 PUFAs

**Figure 6:**
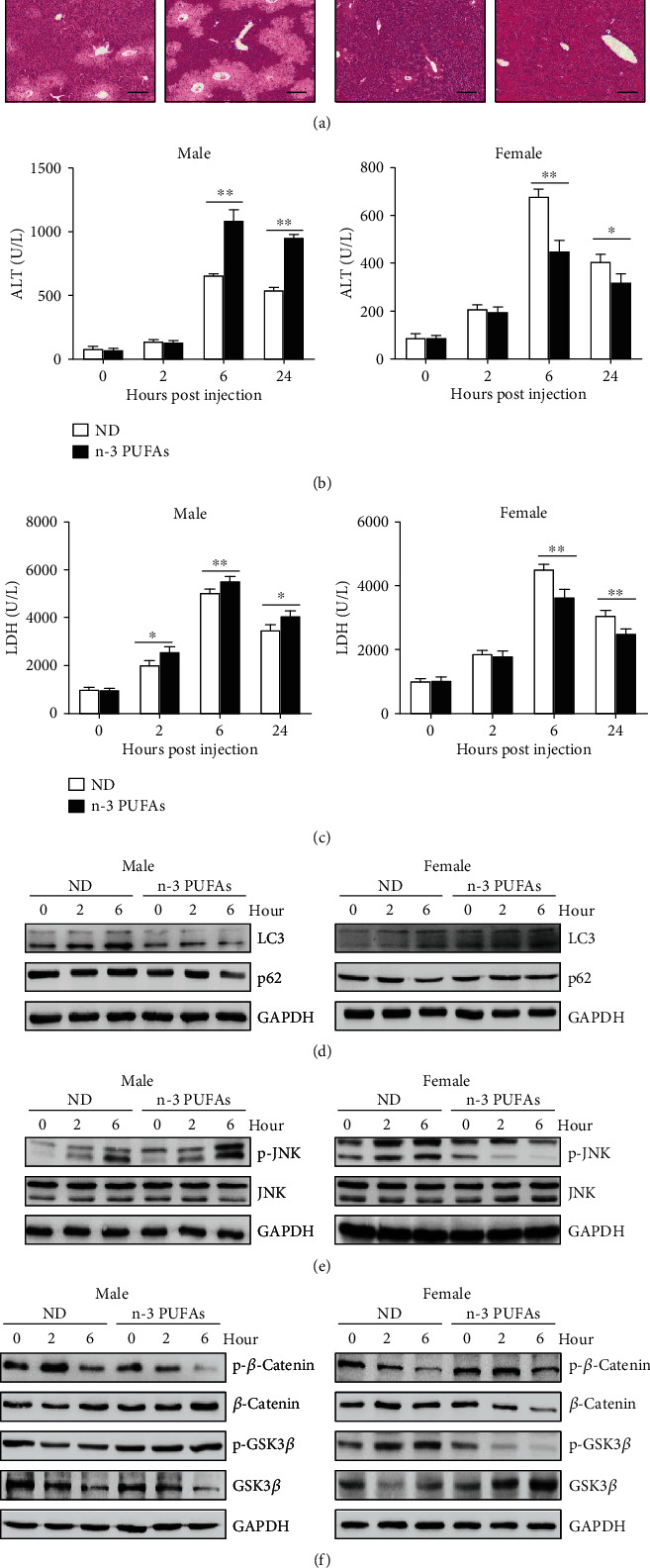
Sex-specific effect of exogenous n-3 PUFAs on APAP-induced liver damage. APAP (400 mg/kg) was intraperitoneally injected into male or female WT mice fed with normal diet or n-3 PUFA-enriched diet (*n* = 5). (a) 24 hours after APAP injection, histological analysis of mouse livers was performed by H&E staining. Scale bars = 100 *μ*m. (b, c) Serum ALT and LDH levels at different time points post-APAP injection were measured. (d) The protein levels of LC3 and p62 in liver tissues were determined by immunoblotting analysis. (e) Phosphorylation of JNK expression in livers was evaluated by immunoblotting analysis. (f) The phosphorylation of *β*-catenin and GSK3*β* was determined by immunoblotting analysis at the indicated time point after APAP administration. ^∗∗^*p* < 0.01. The data represent three independent experiments with similar results.

To better characterize our findings, C57BL/6 mice were fed with n-3 PUFA-enriched diet for 3 weeks, before APAP injection. Similar to *fat-1* mice, n-3 PUFA-fed male mice performed severer liver injury than the control mice during APAP exposure, while female mice with dietary n-3 PUFAs showed attenuated hemorrhage area as implied by H&E staining (Figure 6(a)). Consistently, exogenous n-3 PUFAs elevated serum ALT and LDH activities in male APAP-challenged mice but decreased activities of ALT and LDH in female APAP-administrated mice (Figures 6(b) and 6(c)). Meanwhile, we observed comparable hepatic GSH levels between n-3 PUFA-fed mice and the control mice upon APAP injection (Supplemental Fig. 1C). Besides, upon APAP injection, attenuated expressions of LC3 and p62 were detected in liver tissues from male mice fed with n-3 PUFAs compared to those from the control mice. By contrast, increased hepatic levels of LC3 and p62 were found in female n-3 PUFA-fed mice (Figure 6(d)). Likewise, the livers obtained from n-3 PUFA-fed male mice exhibited aggravated JNK phosphorylation, but n-3 PUFA-treated female mice showed an attenuated hepatic level of phosphorylated JNK against APAP challenge (Figure 6(e)). Moreover, exogenous n-3 PUFA administration promoted GSK3*β* phosphorylation, thereby preventing *β*-catenin phosphorylation in male mice. As a result, the hepatic level of *β*-catenin was higher in n-3 PUFA-fed male mice than in the control mice after APAP injection (Figure 6(f)). By contrast, the expression of *β*-catenin was significantly reduced in liver tissues from n-3 PUFA-fed female mice compared with those from the control mice upon APAP exposure (Figure 6(f)). These data indicate that the exogenous n-3 PUFA supplement exhibits similar effects with endogenous n-3 PUFAs on APAP hepatotoxicity.

## 4. Discussion

n-3 PUFAs have been reported to exhibit a wide effect on liver injury in several animal models [12, 14, 16]. In our current study, we have demonstrated that n-3 PUFAs exert sex-specific control of APAP hepatotoxicity in mice. Our data showed that n-3 PUFAs aggravated APAP-induced liver injury in male mice but ameliorated APAP hepatotoxicity in female mice through differential regulation of autophagy activation. Additionally, we revealed that the GSK3*β*-mediated activation of *β*-catenin signaling is related to the sex-dependent effect of n-3 PUFAs on APAP hepatotoxicity.

Sex differences in the pathogenesis of most chronic liver diseases are evident [35, 36]. Estrogens can reduce drug-induced liver injury in mice [37], while androgen excess drives progression to liver inflammation and increases the risk of NAFLD in women with polycystic ovary syndrome [38]. Zaima et al. observed that dietary intake of n-3 PUFAs increased the serum level of testosterone in mice, suggesting that n-3 PUFAs might modulate testosterone metabolism [39]. Our findings were in line with the previous study which reported that endogenous n-3 PUFAs protect against APAP-induced hepatotoxicity in female mice [40]. Intriguingly, we here observed that n-3 PUFAs aggravated the APAP-induced liver injury in male mice. Moreover, our study showed that estrogen treatment reversed the effect of n-3 PUFAs on the APAP hepatotoxicity in male *fat-1* mice, indicating that estrogen contributes to the sex differences in the regulation of APAP toxicity by n-3 PUFAs. APAP-induced hepatotoxicity is majorly characterized by overwhelmingly increased oxidative stress [41]. The lower susceptibility of female mice is achieved by the enhanced detoxification of ROS related to the accelerated recovery of mitochondrial GSH levels, which ameliorates the subsequent JNK activation and liver damage [42]. Our data also showed that n-3 PUFAs modulated the generation of intercellular ROS and JNK activation in APAP-challenged mice. The differential regulation of APAP hepatotoxicity by n-3 PUFAs in male and female mice was associated with the change of oxidative response.

Autophagy plays a vital role in liver physiology [14, 43] and has been considered as a possible therapeutic target for APAP overdose [44]. Interestingly, it has been reported that ROS inhibited autophagy activation through TRPM2-CAMK2-BECN1P Ca^2+^ influx signaling during APAP hepatoxicity [45]. Our studies found inhibited autophagy activation and aggravated ROS production in male *fat-1* mice but promoted autophagy activation and limited ROS level in female *fat-1* mice. As previously reported, both upregulated LC3 and p62 expressions were observed in cisplatin-induced acute kidney injury, which involved the crosstalk between autophagy and signaling related to oxidative response [46]. We supposed that n-3 PUFAs suppressed autophagy activation, which led to accumulated injured mitochondria, thereby resulting in excessive ROS production during APAP exposure. The enhanced ROS production may in turn inhibit autophagy activation in the damaged hepatocytes. Moreover, estrogen might modulate the n-3 PUFA-mediated regulation of autophagy-oxidative stress interconnection during drug-induced liver injury.

*β*-Catenin plays an essential role in liver regeneration, and the role of *β*-catenin in acute liver injury has been steadily discovered [47]. Jiang et al. reported increased *β*-catenin-aggravated Con A-induced liver damage through regulation of the NF-*κ*B pathway [48]. Loss of *β*-catenin in hepatocytes significantly ameliorated fulminant hepatitis via a similar mechanism [49]. Furthermore, *β*-catenin has been found to inhibit both LC3 and p62 expressions, which indicated impaired autophagosome formation [31]. We found higher levels of *β*-catenin in hepatocytes accompanied by severer liver damage during APAP exposure. Surprisingly, n-3 PUFAs exhibit a distinct effect on *β*-catenin signaling activation between male and female mice with APAP administration. Besides, estrogen did not affect the APAP hepatotoxicity by n-3 PUFA treatment when the Wnt/*β*-catenin signaling was blocked by XAV939, suggesting that the gender-dependent effect of n-3 PUFAs on APAP-induced liver injury relies on the regulation of the Wnt/*β*-catenin signaling pathway. Indeed, the crosstalk between Wnt/*β*-catenin and estrogen receptor signaling has been reported in cell differentiation [50]. It is noteworthy that GSK3 plays a critical role in a diverse range of signaling pathways [33, 51]. GSK3*β* phosphorylation triggers *β*-catenin destabilization which is a key event in Wnt/*β*-catenin signaling [33]. Krishnankutty et al. found that GSK3*β* was principally in the active form with little Ser9 phosphorylation in mouse brains, and the phosphoisotypes of GSK3*β* change related to the regions of the brain, age, sex, and disease conditions [52]. In this study, we determined that GSK3*β* activity is differentially regulated by n-3 PUFAs in the liver depending on sex. Our data suggest that gender disruption in GSK3*β*-mediated Wnt/*β*-catenin pathway activation is linked to the differential regulation of autophagy and oxidative response in male and female n-3 PUFA-enriched mice upon APAP challenge.

In summary, our data revealed the significant differences in n-3 PUFA-regulated APAP hepatotoxicity in male and female mice. We demonstrated that *β*-catenin-mediated regulation of the autophagy process is the essential event for the n-3 PUFA-modulated APAP hepatotoxicity. This work would also provide a better understanding of the role of GSK3*β*/Wnt/*β*-catenin signaling in autophagy activation in relation to the sex-dependent manner of n-3 PUFAs in the regulation of APAP-induced liver injury.

## Data Availability

The data used to support the findings of this study are available from the corresponding author upon request.

## Abbreviations

ALT: Alanine aminotransferase
APAP: Acetaminophen
BSA: Bovine serum albumin
DHA: Docosahexaenoic acid
E2: 17*β*-Estradiol
EPA: Eicosapentaenoic acid
FBS: Fetal bovine serum
GSH: Glutathione
H&E: Hematoxylin and eosin
HRP: Horseradish peroxidase
JNK: c-Jun N-terminal kinase
LC3-I: Cytosolic microtubule-associated protein 1 light chain 3
LDH: Lactate dehydrogenase
n-3 PUFAs: Omega-3 polyunsaturated fatty acids
NAFLD: Nonalcohol fatty liver disease
NAPQI: N-Acetyl-p-benzoquinone imine
PE: Phosphatidylethanolamine
ROS: Reactive oxygen species
WCL: Whole-cell lysate
WT: Wild type.

## Supplementary Materials

Supplementary Materials Supplemental Figure 1: sex-dependent effect of n-3 PUFAs on APAP-induced liver damage did not rely on NAPQI formation. (A) 400 mg/kg of APAP was intraperitoneally injected to male or female WT and fat-1 transgenic mice (*n* = 5). Hepatic GSH levels were measured at the indicated time point after APAP injection. (B) Male WT or fat-1 mice (*n* = 5) were intraperitoneally injected with 100 mg/kg of E2 7 days before APAP (400 mg/kg) administration. Hepatic GSH level was detected after APAP challenge. (C) 400 mg/kg of APAP was intraperitoneally injected into male or female WT mice fed with normal diet or n-3 PUFA-enriched diet (*n* = 5). Hepatic GSH level was determined at the indicated time point. NS: not significant. The data represent three independent experiments with similar results. Supplemental Figure 2: *β*-catenin inhibition abrogated the effect of estrogen on oxidative stress response and autophagy activation during APAP exposure. Primary hepatocytes from male WT or fat-1 mice were isolated and pretreated with 100 nM E2 overnight. Subsequently, the cells were incubated with 2 *μ*M XAV939 for 2 hours and stimulated with APAP for another 6 hours. (A) The ROS level in the cells was detected by flow cytometry labeling with fluorescent probe DCFH-DA at 6 hours post-APAP administration. (B) Phosphorylation of JNK expression was measured by immunoblotting analysis. (C) The levels of LC3 and p62 were evaluated by immunoblotting analysis. ^∗∗^*p* < 0.01; NS: not significant. The data represent three independent experiments with similar results.
