# Supplementary material for: Gender Difference on the Effect of Omega-3 Polyunsaturated Fatty Acids on Acetaminophen-Induced Acute Liver Failure
Source: Oxid Med Cell Longev. 2020 Aug 27;2020:8096847. doi: 10.1155/2020/8096847 (PMC7474378; doi:10.1155/2020/8096847)
Supplement: Supplementary Materials — Supplemental Figure 1: sex-dependent effect of n-3 PUFAs on APAP-induced liver damage did not rely on NAPQI formation. (A) 400 mg/kg of APAP was intraperitoneally injected to male or female WT and fat-1 transgenic mice (n = 5). Hepatic GSH levels were measured at the indicated time point after APAP injection. (B) Male WT or fat-1 mice (n = 5) were intraperitoneally injected with 100 mg/kg of E2 7 days before APAP (400 mg/kg) administration. Hepatic GSH level was detected after APAP challenge. (C) 400 mg/kg of APAP was intraperitoneally injected into male or female WT mice fed with normal diet or n-3 PUFA-enriched diet (n = 5). Hepatic GSH level was determined at the indicated time point. NS: not significant. The data represent three independent experiments with similar results. Supplemental Figure 2: β-catenin inhibition abrogated the effect of estrogen on oxidative stress response and autophagy activation during APAP exposure. Primary hepatocytes from male WT or fat-1 mice were isolated and pretreated with 100 nM E2 overnight. Subsequently, the cells were incubated with 2 μM XAV939 for 2 hours and stimulated with APAP for another 6 hours. (A) The ROS level in the cells was detected by flow cytometry labeling with fluorescent probe DCFH-DA at 6 hours post-APAP administration. (B) Phosphorylation of JNK expression was measured by immunoblotting analysis. (C) The levels of LC3 and p62 were evaluated by immunoblotting analysis. ∗∗p < 0.01; NS: not significant. The data represent three independent experiments with similar results. [file 8096847.f1.docx]

**Supplemental Figures**


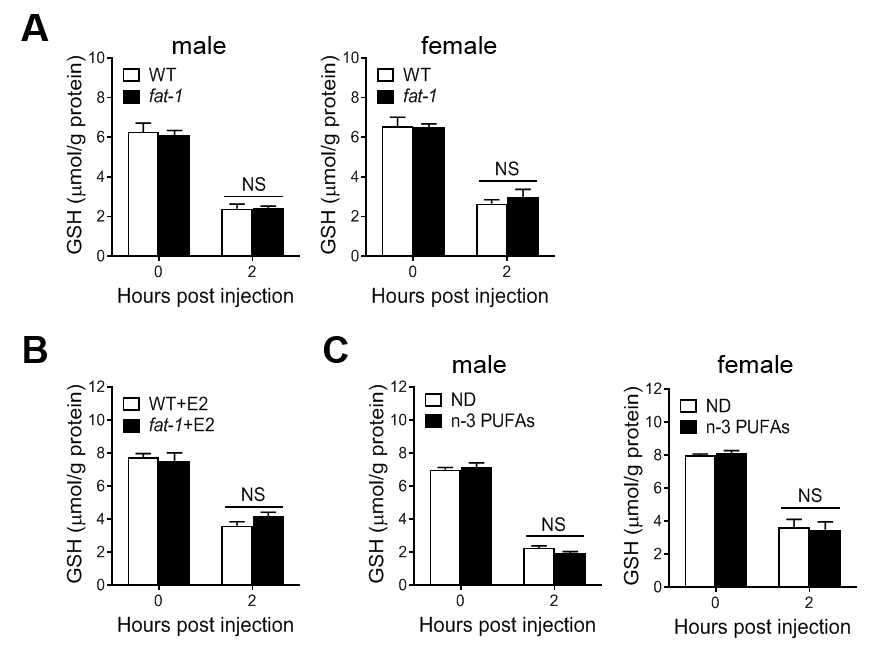


**Supplemental Fig. 1. Sex-dependent effect of n-3 PUFAs on APAP-induced liver damage did not rely on NAPQI formation.** A. 400mg/kg of APAP was intraperitoneally injected to male or female WT and *fat-1* transgenic mice (n=5). Hepatic GSH levels were measured at the indicated time point after APAP injection. B. Male WT or *fat-1* mice (n=5) were intraperitoneally injected with 100mg/kg of E2 7 days before APAP (400mg/kg) administration. Hepatic GSH level was detected after APAP challenge. C. 400mg/kg of APAP was intraperitoneally injected into male or female WT mice fed with normal diet or n-3 PUFAs-riched diet (n=5). Hepatic GSH level was determined at the indicated time point. NS, not significant. The data represent three independent experiments with similar results.


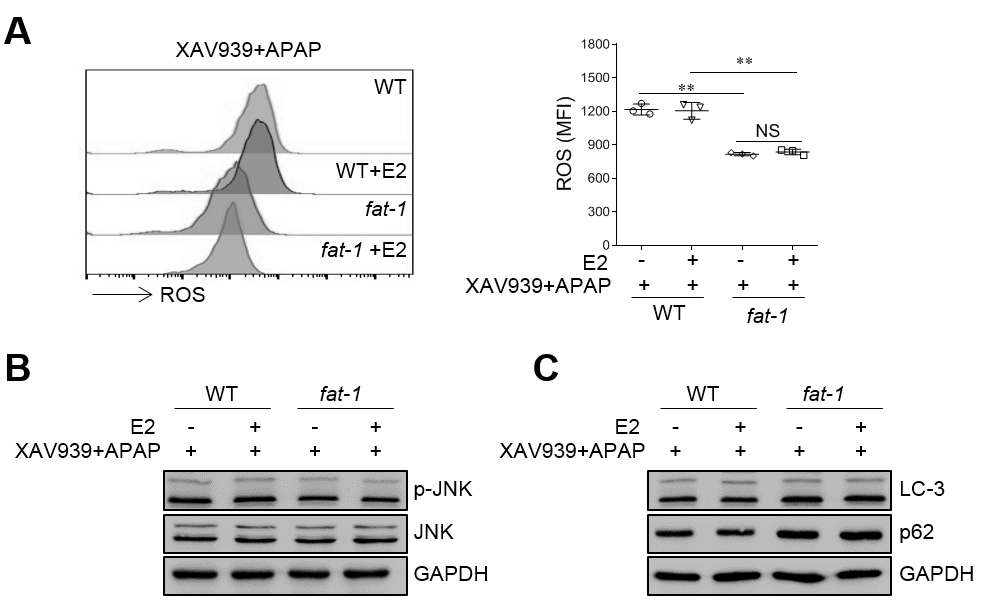


**Supplemental Fig. 2. β-catenin inhibition abrogated the effect of estrogen on oxidative stress response and autophagy activation during APAP exposure.** Primary hepatocytes from male WT or *fat-1* mice were isolated and pretreated with 100nM E2 for overnight. Subsequently, the cells were incubated with 2μM XAV939 for 2 hours, and stimulated with APAP for another 6 hours. A. The ROS level in the cells was detected by flow cytometry labeling with fluorescent probe DCFH-DA at 6 hours post APAP administration. B. Phosphorylation of JNK expression was measured by immunoblotting analysis. C. The levels of LC3 and p62 were evaluated by immunoblotting analysis. ***p*<0.01, NS, not significant. The data represent three independent experiments with similar results.
